# Long-Term Exercise Is a Potent Trigger for ΔFosB Induction in the Hippocampus along the dorso–ventral Axis

**DOI:** 10.1371/journal.pone.0081245

**Published:** 2013-11-25

**Authors:** Takeshi Nishijima, Masashi Kawakami, Ichiro Kita

**Affiliations:** Laboratory of Behavioral Physiology, Graduate School of Human Health Sciences, Tokyo Metropolitan University, Tokyo, Japan; Karolinska Inst, Sweden

## Abstract

Physical exercise improves multiple aspects of hippocampal function. In line with the notion that neuronal activity is key to promoting neuronal functions, previous literature has consistently demonstrated that acute bouts of exercise evoke neuronal activation in the hippocampus. Repeated activating stimuli lead to an accumulation of the transcription factor ΔFosB, which mediates long-term neural plasticity. In this study, we tested the hypothesis that long-term voluntary wheel running induces ΔFosB expression in the hippocampus, and examined any potential region-specific effects within the hippocampal subfields along the dorso–ventral axis. Male C57BL/6 mice were housed with or without a running wheel for 4 weeks. Long-term wheel running significantly increased FosB/ΔFosB immunoreactivity in all hippocampal regions measured (i.e., in the DG, CA1, and CA3 subfields of both the dorsal and ventral hippocampus). Results confirmed that wheel running induced region-specific expression of FosB/ΔFosB immunoreactivity in the cortex, suggesting that the uniform increase in FosB/ΔFosB within the hippocampus is not a non-specific consequence of running. Western blot data indicated that the increased hippocampal FosB/ΔFosB immunoreactivity was primarily due to increased ΔFosB. These results suggest that long-term physical exercise is a potent trigger for ΔFosB induction throughout the entire hippocampus, which would explain why exercise can improve both dorsal and ventral hippocampus-dependent functions. Interestingly, we found that FosB/ΔFosB expression in the DG was positively correlated with the number of doublecortin-immunoreactive (i.e., immature) neurons. Although the mechanisms by which ΔFosB mediates exercise-induced neurogenesis are still uncertain, these data imply that exercise-induced neurogenesis is at least activity dependent. Taken together, our current results suggest that ΔFosB is a new molecular target involved in regulating exercise-induced hippocampal plasticity.

## Introduction

Exercise confers diverse benefits on molecular, structural, and functional aspects of the hippocampus in rodents [1,2], some of which were supported by human studies [3,4]. However, the mechanisms underlying the exercise-induced changes in hippocampal plasticity are not sufficiently understood. Previous literature has consistently demonstrated that exercise evokes hippocampal neuronal activation in rodents. Immunohistochemical studies using c-Fos, a marker of transient neuronal activation, have demonstrated that both forced and voluntary running increased c-Fos expression in the dentate gyrus (DG), CA1, and CA3 subfields of the rodent hippocampus [5-7]. In addition, a previous study using laser-Doppler flowmetry (LDF) has demonstrated that mild treadmill running increased regional cerebral blood flow (rCBF), an alternative marker of neuronal activation, in the CA1 subfield in rat [8]. Immunohistochemical studies enable detailed region-specific analyses after exercise has ceased, while LDF enables real-time monitoring of rCBF in a localized area during exercise. Despite the advantages and limitations of each study, these studies similarly demonstrated an effect of acute bouts of exercise on hippocampal neuronal activity. These results suggest a mechanism whereby long-term regular exercise promotes hippocampal plasticity by repeatedly triggering neuronal activation [9].

The transcription factor ΔFosB, a truncated splice isoform of full-length FosB, is induced by various types of repeated stimuli in specific brain regions, where it gradually accumulates because of its unique stability (a half-life of weeks) [10-12]. A growing body of evidence demonstrates that increased levels of ΔFosB mediate long-lasting neural and behavioral plasticity associated with particular stimuli [11,13]. For example, chronic administration of drugs of abuse such as cocaine and morphine commonly increases ΔFosB expression in the nucleus accumbens, representing one of the molecular mechanisms underlying increased sensitivity to these drugs [11,14,15]. Similarly to other reward stimuli, including high-fat diet and sexual experience [16,17], long-term voluntary wheel running also increased FosB/ΔFosB immunoreactivity in rat nucleus accumbens, suggesting that voluntary running is a natural reward for rodents [18,19]. However, to the best of our knowledge, no literature has examined whether repeated exposure to physical exercise induces ΔFosB expression in the hippocampus. Because exercise triggers neuronal activation in the hippocampus, we hypothesized that long-term voluntary wheel running would also induce ΔFosB expression in the hippocampus. While the exact mechanisms by which ΔFosB regulates hippocampal plasticity remain uncertain, studies have demonstrated that mice lacking the *fosB* gene show impaired hippocampal neurogenesis and increased depression-like behavior [20,21]. Indeed, exercise is known to enhance neurogenesis and have antidepressant properties [22-25]. If our hypothesis is correct, ΔFosB would be a new potential molecular target mediating exercise-induced hippocampal plasticity.

The hippocampus has anatomical and functional gradient along its longitudinal (dorso–ventral) axis [26]. The dorsal hippocampus plays a key role in spatial learning and memory [27,28], whereas the ventral hippocampus is preferentially involved in regulating emotional behaviors [29,30]. Furthermore, studies have demonstrated that physiological stimuli induce different patterns of c-Fos expression in the dorsal and ventral portions of the hippocampus [31-33]. Because exercise improves both dorsal [34-37] and ventral hippocampus-dependent functions [24,25,38], it is important to examine whether long-term voluntary running causes region-specific expression of ΔFosB in the hippocampus.

The primary hypothesis of this study was that long-term voluntary wheel running would induce ΔFosB expression in the mouse hippocampus. This hypothesis was investigated by FosB/ΔFosB immunohistochemistry in the dorsal and ventral hippocampal subfields, DG, CA1, and CA3, with extra emphasis on identifying region-specific induction. Results were confirmed by western blotting, which was used to identify the isoform of *fosB* gene products induced in the hippocampus. We also examined the cortex for region-specific FosB/ΔFosB induction to rule out the possibility that long-term exercise non-specifically increased FosB/ΔFosB immunoreactivity in the brain. Finally, the correlative association between FosB/ΔFosB expression and neurogenesis was investigated as the first step in seeking the functional implications of exercise-induced ΔFosB induction in regulating hippocampal plasticity.

## Materials and Methods

### 1: Animals and ethics statement

Twenty male C57BL/6 mice (8 weeks of age) were purchased from a commercial breeder (SLC, Shizuoka, Japan). Ten mice were used for Experiment 1, and the other ten for Experiment 2. Mice were housed under controlled conditions of temperature (22–24°C) and light (12/12-h light/dark cycle, light on at 0500), and were provided food and water *ad libitum*. All experimental procedures were approved by the Animal Experimental Ethics Committee of the Tokyo Metropolitan University.

In each experiment, upon arrival, mice were randomly assigned to either a control group (Control, n = 5) or a running group (Runner, n = 5). During the first week, all mice were housed in standard plastic cages in groups (5 mice/cage) for initial acclimatization. Then, Runner mice were transferred into a cage equipped with a running wheel (ENV-046, Med Associate Inc., Georgia, VT, USA). Because social isolation is known to suppress exercise-induced neurogenesis in the hippocampus [39], Runner mice were housed as a group (5 mice/cage) for an additional 4 weeks. The number of wheel rotations was recorded each morning and body weight (g) was measured weekly.

### 2: Experiment 1. Immunohistochemical examination of FosB/ΔFosB expression and hippocampal neurogenesis

#### 2.1: Perfusion and tissue processing

The morning (0900–1100) after the last day of the running period, the mice were deeply anesthetized with pentobarbital sodium and transcardially perfused with cold saline. The brain was quickly removed and post-fixed in 4% paraformaldehyde in 0.1 M phosphate buffered saline (PBS, pH 7.4) overnight. The brain was then cryoprotected in 30% sucrose in PBS and frozen until further processing. Coronal brain sections (40 μm) of a hemisphere were obtained using a freezing microtome and collected in PBS with 0.01% sodium azide.

#### 2.2: Immunohistochemistry

A one-in-six series of sections was randomly selected for FosB/ΔFosB immunostaining. An adjacent series was used for labeling doublecortin (DCX), a marker of immature neurons validated for assessing neurogenesis [40,41]. After quenching endogenous peroxidase activity with 1% H_2_O_2_ in PBS, free-floating sections were preincubated with blocking solution containing 10% normal horse serum in PBS for 2 h. Following rinses in PBS, sections were incubated with rabbit polyclonal pan-FosB antibody (1:1000, sc-48, Santa Cruz Biotechnology, Dallas, TX, USA) diluted in PBS with 0.5% Triton X-100 and 0.5% BSA (PBST-BSA) for 24 h at 4°C. Another series of sections were incubated with goat polyclonal anti-DCX antibody (1:500, sc-8066, Santa Cruz) in PBST-BSA for 48 h at 4 °C. The sections were further incubated with an appropriate biotinylated secondary antibody (anti-rabbit IgG, 1:1000, AP182B; anti-goat IgG, 1:1000, AP180B, both antibodies from EMD Millipore, Billerica, MA, USA) in PBST-BSA for 2 h at room temperature. The sections were then treated with avidin-biotin-peroxidase complex (Vectastain ABC peroxidase kit, Vector Laboratories Inc, Burlingame, CA, USA) for 90 min following manufacturer’s instructions. The antigens were finally visualized with 0.02% 3,3-diaminobenzidine (DAB) in 0.1 M Tris-HCl (pH 7.6) containing 0.01% H_2_O_2_. For FosB/ΔFosB immunostaining, the reaction was intensified with nickel ammonium sulfate. For DCX staining, cell nuclei were counterstained with Nissl staining. Sections were mounted onto gelatin-coated slides and coverslips were placed.

#### 2.3: Quantification of FosB/ΔFosB immunoreactivity using image thresholding

The pan-FosB antibody used in this study was raised against an internal region shared by FosB and ΔFosB N-terminal region, so that cannot discriminate between the two isoforms. Therefore, the immunostained structures were described as FosB/ΔFosB immunoreactive (FosB/ΔFosB-ir) nuclei. For an unbiased blind quantification, slides were coded prior to analysis. The mouse brain atlas [42] was used to identify location of the following regions of interest (ROIs): granule cell layer (GCL) of DG (3 sections), pyramidal cell layer of CA1 (3 sections) and CA3 (2–3 sections) in the dorsal hippocampus (closed to -2.2 mm from the bregma); DG (2 sections), CA1 (2 sections), and CA3 (2 sections) in the ventral hippocampus (closed to -3.4 mm from the bregma) (Figure 4, left). The caudal sections contain both the dorsal and ventral portions of the hippocampus, but the ventral portion was targeted. In the DG, suprapyramidal (DGsp) and infrapyramidal (DGip) blades were analyzed separately. Motor cortex (2–3 sections, closed to -0.6 mm from the bregma), somatosensory barrel cortex (2–3 sections, closed to -0.6 mm from the bregma), visual cortex (3 sections, closed to -2.9 mm from the bregma), auditory cortex (3 sections, closed to -2.9 mm from the bregma), and olfactory bulb (3 sections, closed to +4.3 mm from the bregma) were also analyzed (Figure 6, left).

**Figure 1:**
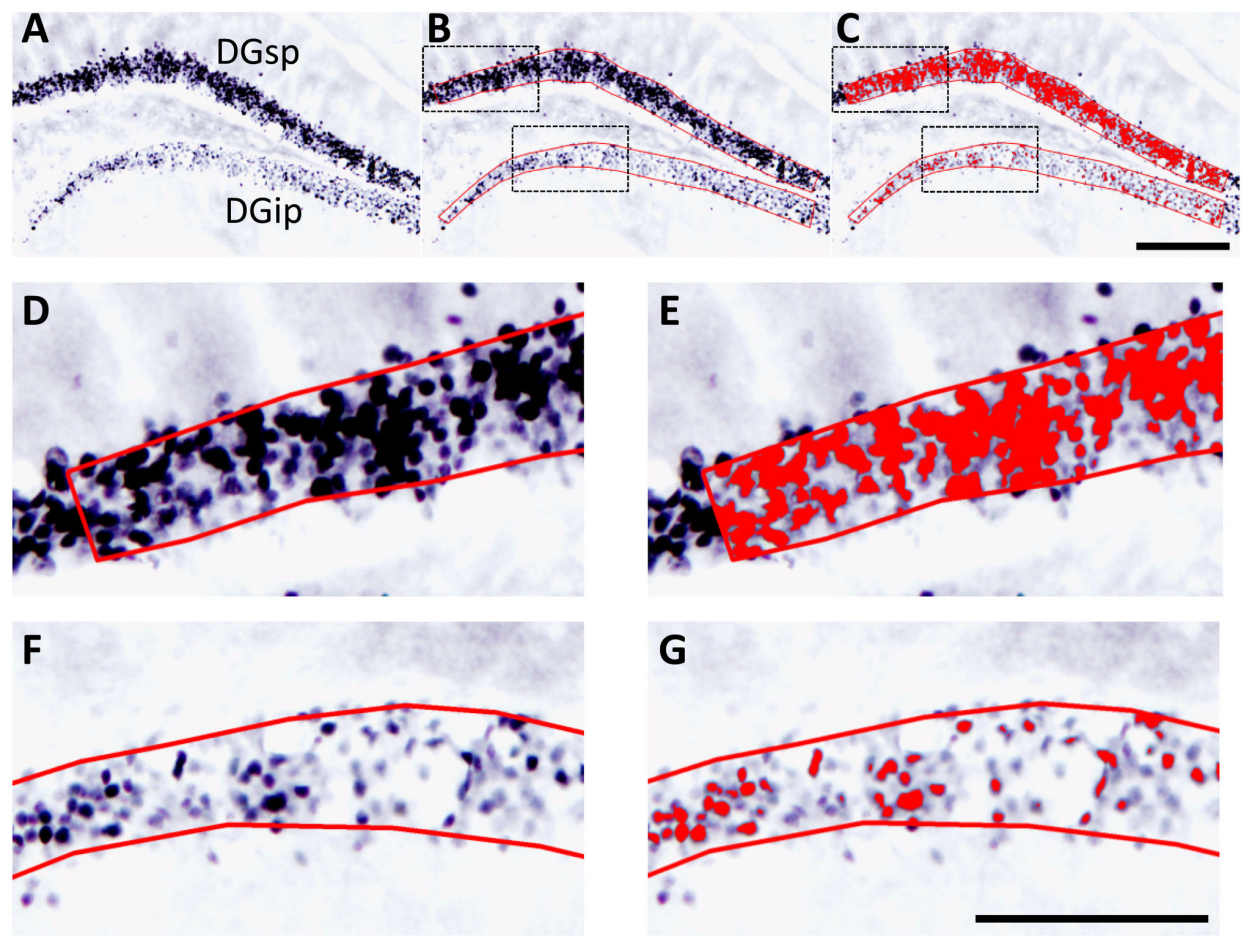
Representative images illustrating the steps involved in image thresholding analysis of FosB/ΔFosB immunoreactivity. (A) An unprocessed image of FosB/ΔFosB-ir nuclei in the dorsal DG. (B) ROIs were selected by overlaying an irregularly shaped polygon (shown in red). Boxes (black, broken lines) represent the areas of magnification shown in (D, top) and (F, bottom). (C) The image was then thresholded, which converted the FosB/ΔFosB-ir nuclei to a red color. Boxes (black, broken lines) represent the areas of magnification shown in (E, top) and (G, bottom). Scale bar in C = 200 μm. (D & E) Magnified images representing dense FosB/ΔFosB expression in the suprapyramidal blade of the DG (DGsp), from (B) and (C), respectively. (F & G) Magnified images representing weak FosB/ΔFosB immunoreactivity in the infrapyramidal blade of the DG (DGip), from (B) and (C), respectively. Scale bar in G = 100 μm.

Digital images (2070 × 1548 pixels) of each ROI were taken using an optical microscope (BX-51, Olympus, Tokyo, Japan) equipped with a CCD camera (DP-73, Olympus) and imaging software (cellSens, Olympus).The objective lens magnification was 10× for hippocampal ROIs and 4× for cortical ROIs. In order to identify moderate-to-strong FosB/ΔFosB immunoreactivity (Figure 1D–G), using several sections in advance, both image acquisition settings (light intensity, size of field stop, exposure time, and white balance) and threshold levels for each of the RGB components were optimized for hippocampal and cortical ROIs. The following analysis was then performed under the optimized conditions (1). ROIs were selected by an irregularly shaped polygon (Figure 1A, B) (2). The image was thresholded, which converted the FosB/ΔFosB-ir nuclei to a red color (Figure 1C-G) (3). The %ROI was then automatically calculated as follows: %ROI = (converted area (in red)/total ROI area) × 100.

To validate this image thresholding analysis, 20 regions were randomly selected from different brain areas with different region sizes. In addition of the image thresholding quantification, the number of FosB/ΔFosB-ir nuclei within the selected regions was manually counted and the density of FosB/ΔFosB-ir nuclei was obtained by dividing the number of FosB/ΔFosB-ir nuclei by the measured area (mm^2^).

#### 2.4: Quantification of DCX-ir immature neurons in the dentate gyrus

The DCX-ir immature neurons in the DG of Runner mice were abundant and overlapping, making it difficult to precisely count the discrete number of DCX-ir soma using an optical microscope. However, in a previous study, Sholl analysis for morphological evaluation showed that each DCX-ir neuron has, on average, a single dendrite when measured within 40 μm of the soma [43]. Therefore, the following original analysis was developed to enable region-specific quantification of DCX-ir neurons.

(1) An image of the GCL was projected on a computer display using imaging software and a 40× objective lens (2). On the live image, a line segment (150 ± 0.1 μm) was drawn along the middle of the GCL (Figure 2) (3). Changing the focal depth, the number of times the line segment crossed DCX-ir dendrites were counted (4). The ROIs (dorsal DGsp, dDGsp; dorsal DGip, dDGip; ventral DGsp, vDGsp; ventral DGip, vDGip) corresponded to the regions where FosB/ΔFosB immunoreactivity was analyzed (5). In every ROI, 2–3 line segments were drawn per section and the number of crossings was averaged over 2–3 sections per mouse. Because the thickness of the GCL is approximately 60–80 μm, the number of crossings should reflect the number of DCX-ir neurons within the restricted region analyzed.

### 3. Experiment 2. Identification of the FosB/ΔFosB isoform induced by wheel running

**Figure 2:**
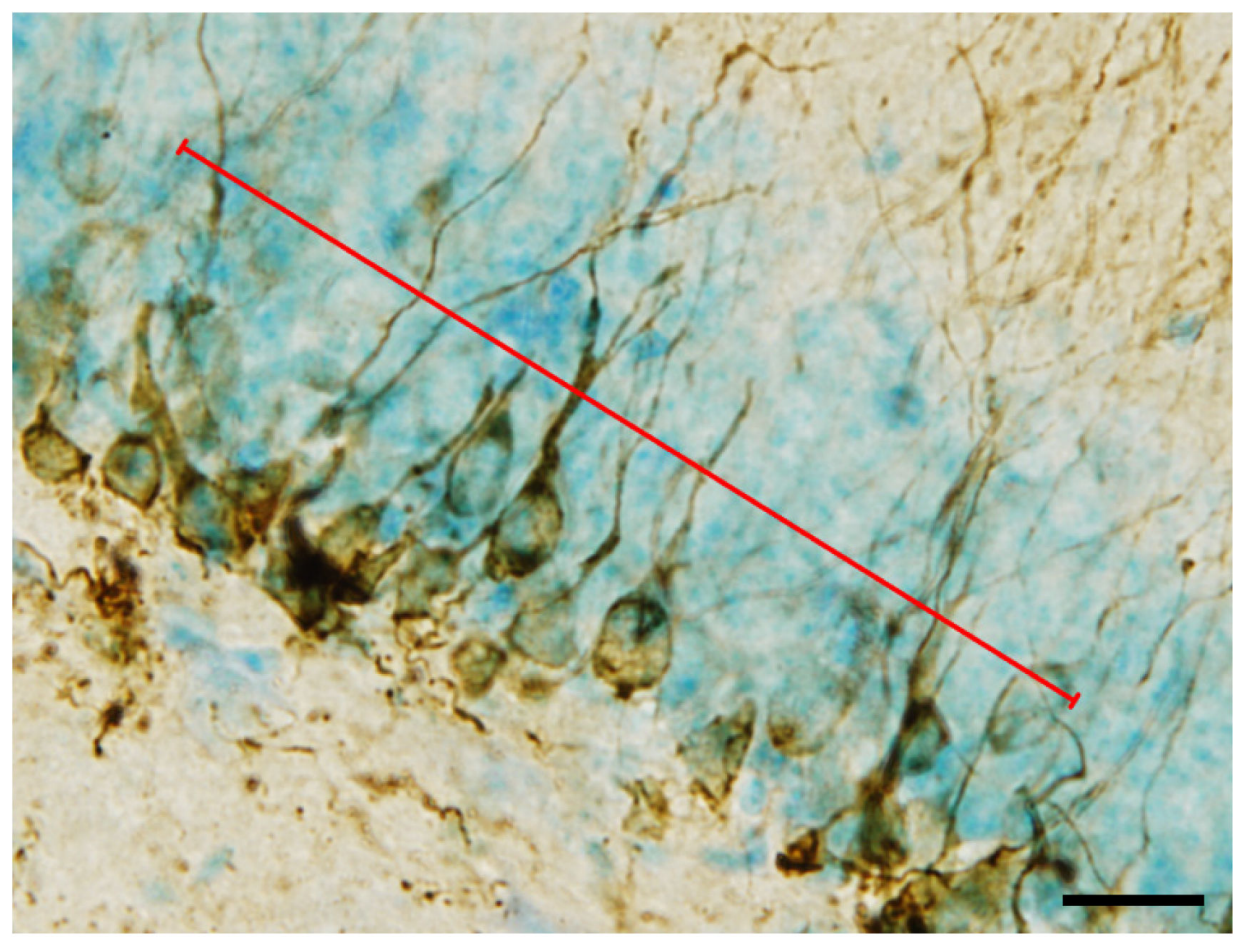
A representative image of DCX-ir immature neurons and a line segment (150 ± 0.1 μm) overlaid for counting the number of crossings with DCX-ir dendrites. Scale bar = 20 μm.

#### 3.1: Perfusion and tissue processing

An additional cohort of mice was treated as above in Experiment 1. After 4 weeks of running intervention, the mice were transcardially perfused with cold saline under deep anesthesia. The hippocampus was quickly dissected out and frozen with liquid nitrogen, and stored at -80°C. The hippocampi of each mouse were homogenized in RIPA buffer (150 mM NaCl, 25 mM Tris-HCl pH 7.6, 1% NP-40, 1% sodium deoxycholate, 0.1% SDS, #8990, Thermo Scientific, IL, USA) containing protease inhibitors (cOmplete Mini, Roche, Manheim, Germany). The lysates were centrifuged for 15 min at 5000 rpm at 4°C and supernatants were collected. Protein concentrations were measured with a BCA Protein Assay kit (#23227, Thermo Scientific, IL, USA).

#### 3.2: Western blotting

Equal amounts of protein (30 μg/lane) were electrophoresed on a 10% polyacrylamide gel, then transferred to a PVDF membrane (Immun-Blot, 0.2 μm, Bio-Rad, MD, USA). Nonspecific binding was blocked by preincubating the membrane for 1 h in TBST (0.5 M NaCl, 20 mM Tris-HCl pH 7.5, 0.1% Tween-20) containing 3% BSA. The membrane was incubated with the pan-FosB antibody (1:1000) that used above for immunohistochemistry, dissolved in TBST containing 3% BSA. Following washes with TBST, the membrane was incubated with HRP-conjugated anti-rabbit IgG antibody (1:5000 in TBST, NA934, GE Healthcare, Buckinghamshire, UK) for 1 h at room temperature. After washes with TBST, protein bands were visualized by incubation with Enhanced Chemiluminescence (Western Lightning Plus-ECL, PerkinElmer, MA, USA) and captured using an Image Quant LAS 4000 mini (GE Healthcare, Buckinghamshire, UK). The membrane was then reprobed with anti-glyceraldehyde-3-phosphate dehydrogenase (GAPDH) antibody (#2275, 1:5000 in TBS-T, Trevigen, MD, USA) as a loading control. The optical density of the protein bands was quantified using Image-J and normalized to the level of GAPDH.

### 4: Statistical analysis

Changes in mouse body weight were analyzed by two-way repeated-measures ANOVA (group × time). An unpaired t-test was used to determine statistical differences between groups (Control vs. Runner). Pearson’s correlation analysis was used to validate the FosB/ΔFosB immunoreactivity analysis (manual counting vs. image thresholding), and to examine the association between the level of FosB/ΔFosB expression and the number of DCX crossings in the DG. Data were presented as mean ± SEM. The threshold for statistical significance was set at *P* < 0.05.

## Results

### 1: Body weight and running distance in Experiments 1 and 2

**Figure 3:**
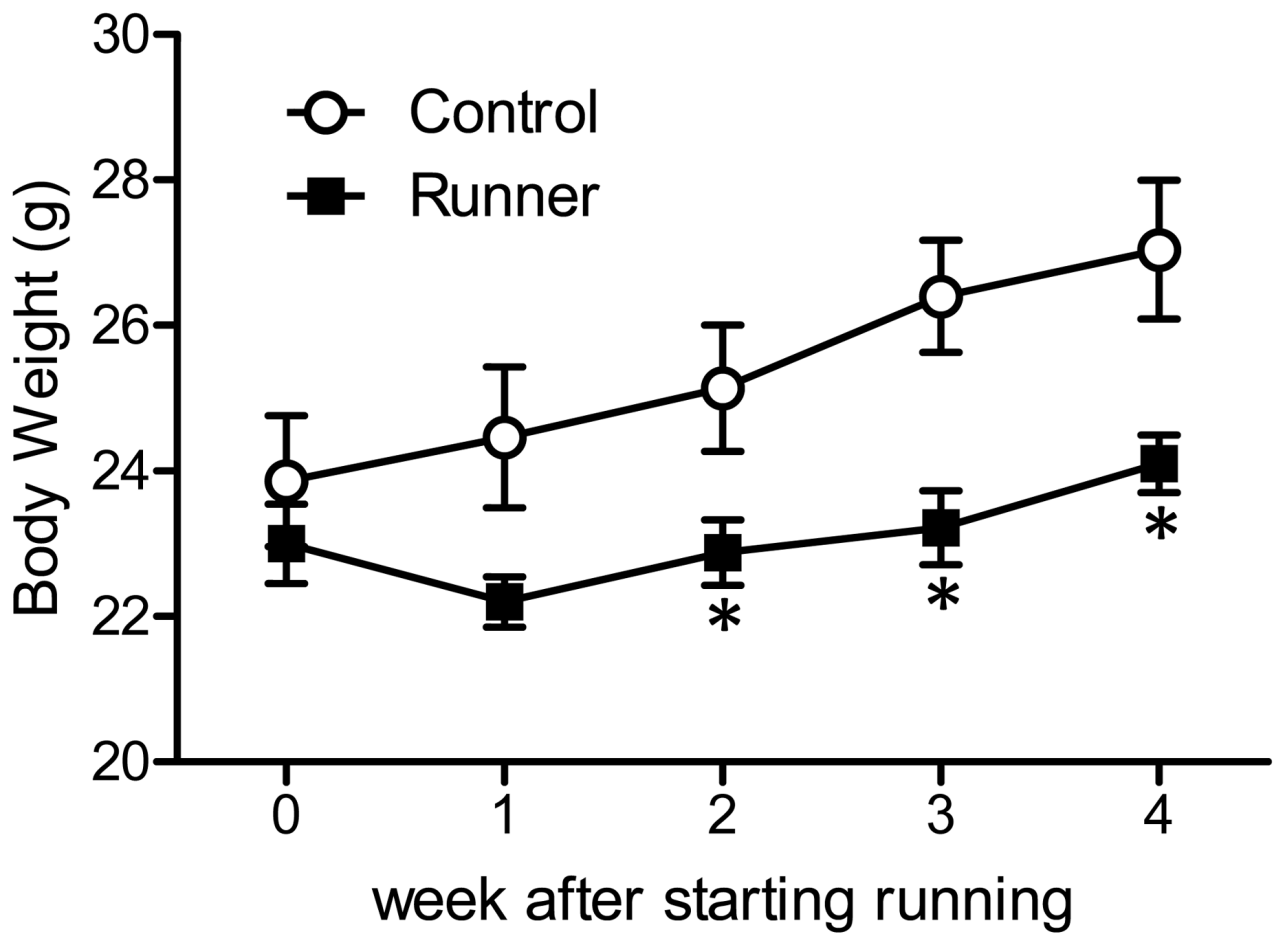
Changes in body weights of Control and Runner mice of Experiment 1 and 2. Data are presented as mean ± SEM (n = 10 per group).

Changes in body weight of both Control and Runner mice in Experiments 1 and 2 are pooled and shown in Figure 3. Two-way repeated-measures ANOVA indicated a significant interaction (group × time, *F*(4, 72) = 13.6, *P* < 0.001) and main effect of group *F*(1, 18) = 6.07, *P* < 0.05), indicating a significantly lower body weight in Runner mice. The running distance per cage is shown in Table 1. Although the precise running distance of each mouse was uncertain because the mice were housed together, regular observation confirmed that all mice frequently performed wheel running. The Runner mice in Experiment 2 ran longer than those in Experiment 1, but the mean running distance (m/day/cage) was consistent throughout each experiment.

**Table 1:** Averaged daily running distance for each week during the 4-weeks running period.

Week after starting running		1	2	3	4
Experiment 1	m/day/cage	16135	17346	17688	17970
Experiment 2	m/day/cage	17073	25666	25967	25941

### 2: Validation of FosB/ΔFosB immunoreactivity quantification using image thresholding

**Figure 4:**
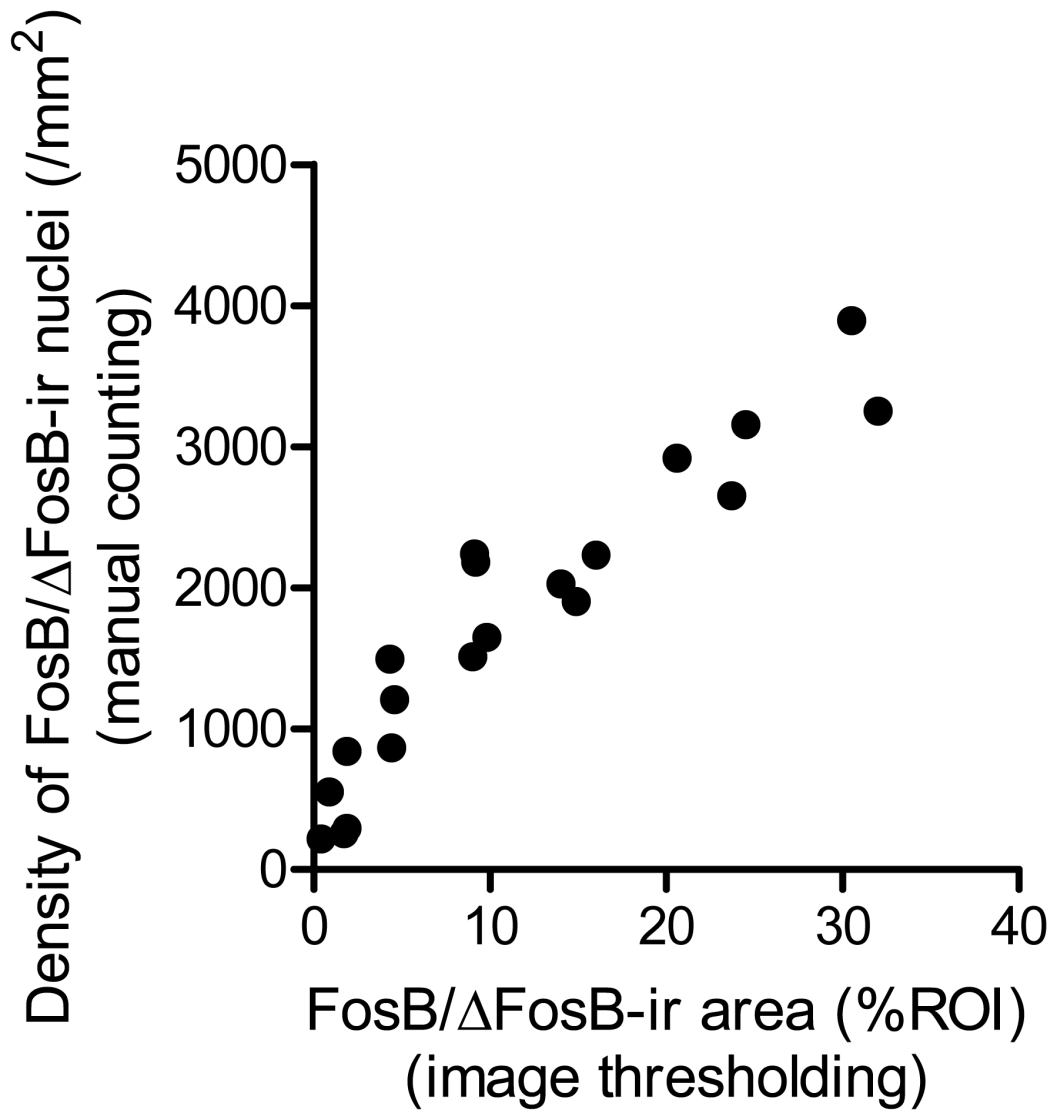
A significant correlation was found between FosB/ΔFosB-ir area (%ROI) obtained by image thresholding and density of FosB/ΔFosB-ir nuclei (nuclei/mm^2^) obtained by manual counting.

There was a significant correlation between FosB/ΔFosB-ir area obtained by image thresholding and density of FosB/ΔFosB-ir nuclei obtained by manual counting (r = 0.941, *P* < 00001, Figure 4).

### 3: FosB/ΔFosB immunoreactivity in the hippocampus

**Figure 5:**
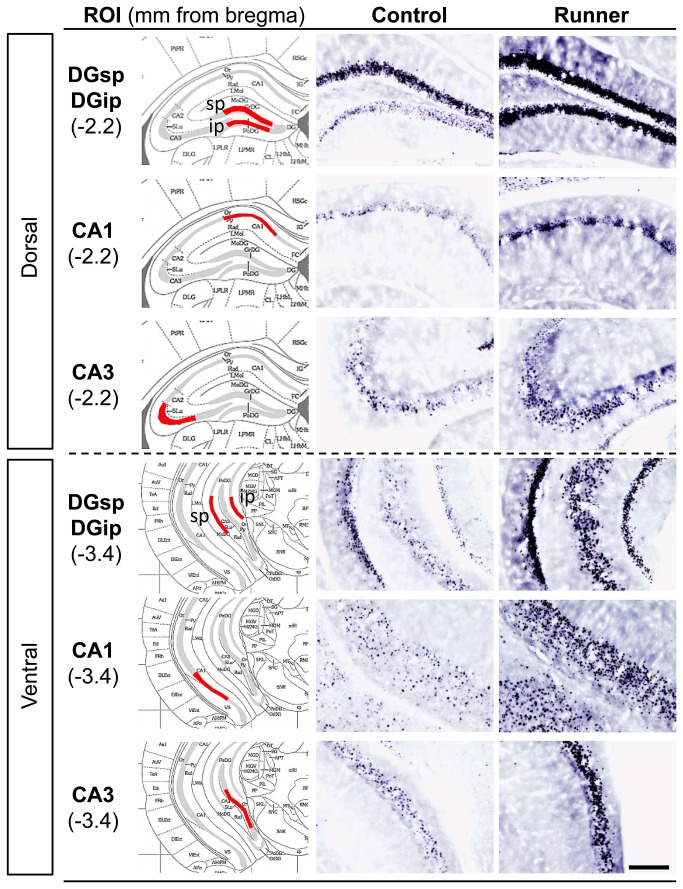
Representative images of FosB/ΔFosB immunostaining in the dorsal and ventral hippocampal ROIs. Left: Illustration adopted from the mouse brain atlas [42]. ROIs measured are indicated in red. Center: Images taken from the brains of Control mice. Right: Images taken from the brains of Runner mice. Scale bar = 200 μm.

**Figure 6:**
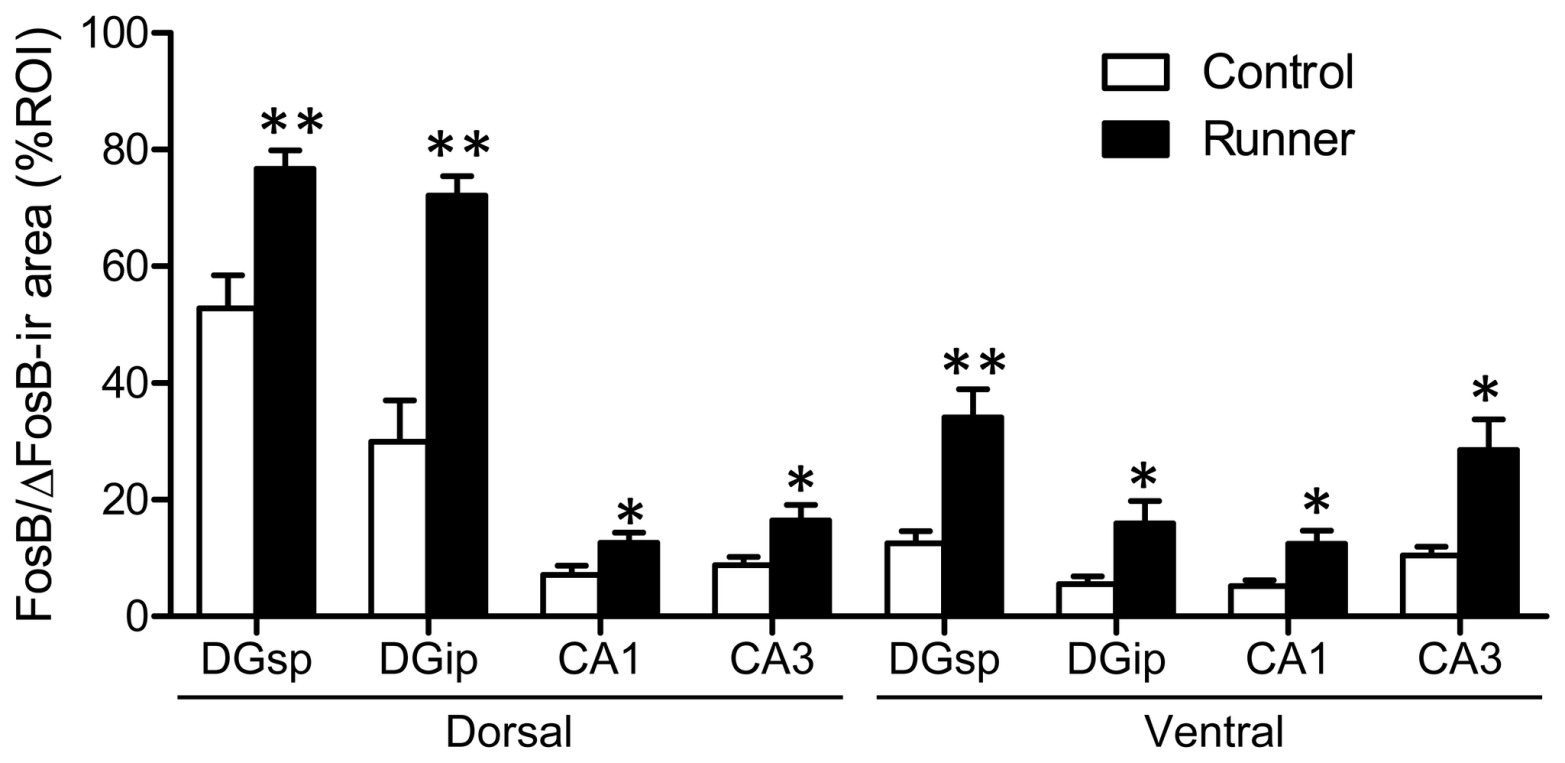
Quantification of FosB/ΔFosB-ir area in the hippocampal ROIs. FosB/ΔFosB-ir area (%ROI) was significantly higher in Runner mice for all ROIs measured in the hippocampus. Data are presented as mean ± SEM (n = 5 per group). * *P* < 0.05, ** *P* < 0.01 vs. Control.

Representative images of FosB/ΔFosB immunostaining in the dorsal and ventral hippocampal subfields were shown in Figure 5. In the all ROIs analyzed, FosB/ΔFosB immunoreactivity in Runner mice (Figure 5, right) was qualitatively higher than that in Control mice (Figure 5, center). In Runner mice, quantitative analysis indicated a significant increase in FosB/ΔFosB-ir area in both the dorsal (DGsp: *P* < 0.01; DGip: *P* < 0.01; CA1: *P* < 0.05; CA3: *P* < 0.05) and the ventral hippocampal subfields (DGsp: *P* < 0.01; DGip: *P* < 0.05; CA1: *P* < 0.05; CA3: *P* < 0.05; Figure 6).

### 4: FosB/ΔFosB immunoreactivity in the cortex

**Figure 7:**
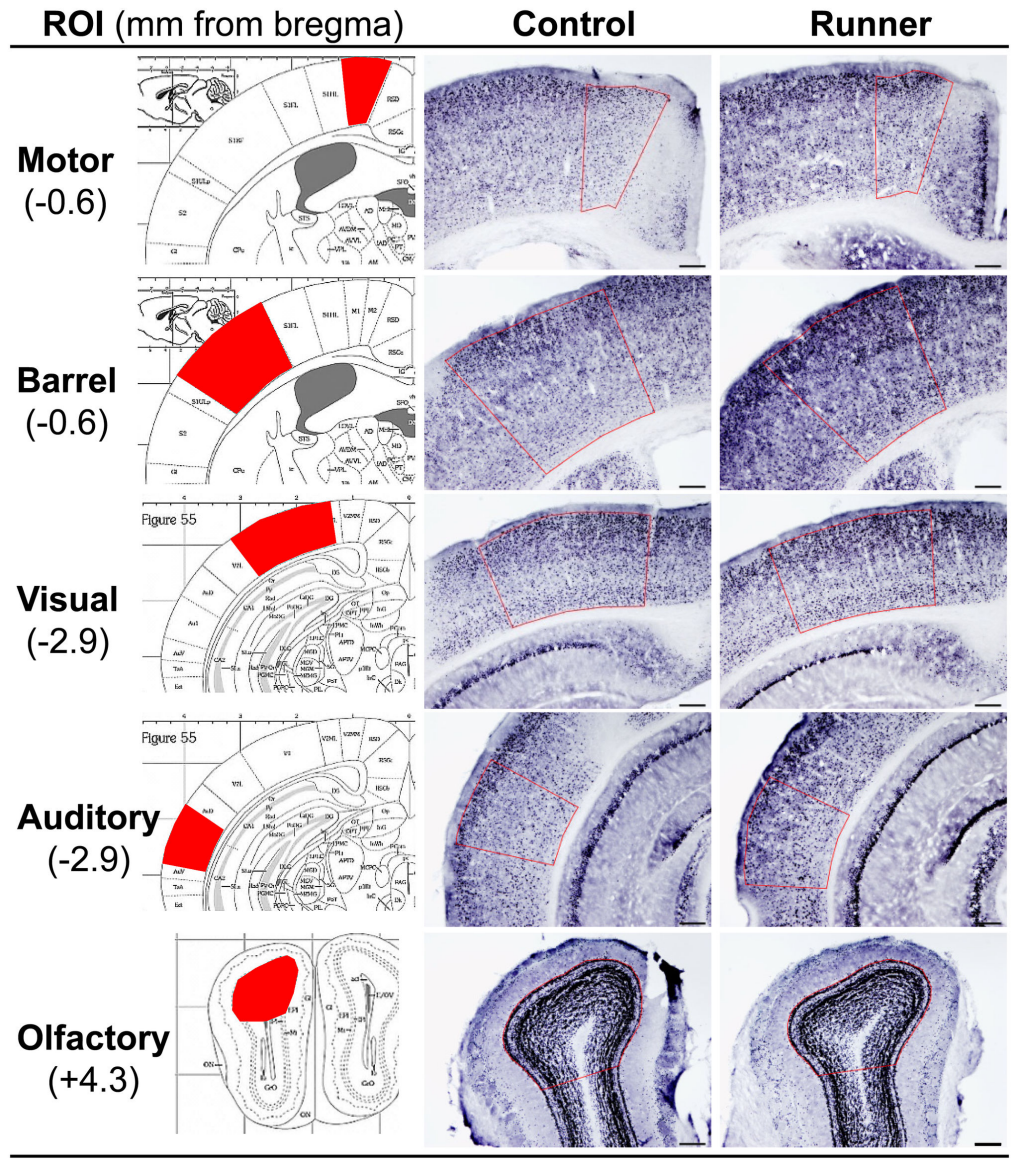
Representative images of FosB/ΔFosB immunostaining in the cortical ROIs. Left: Illustration adopted from the mouse brain atlas [42]. ROIs measured are indicated in red. Center: Images taken from the brains of Control mice. Right: Images taken from the brains of Runner mice. Scale bar = 200 μm.

**Figure 8:**
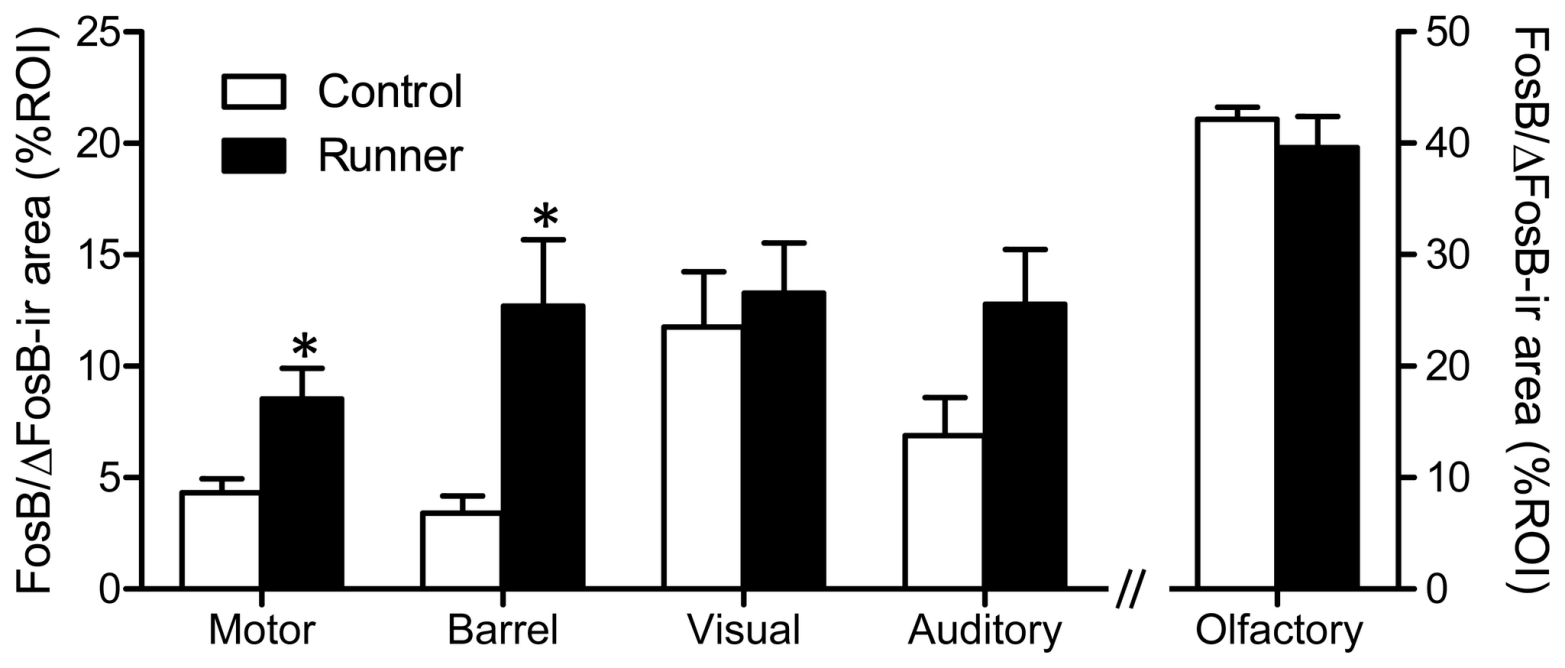
Quantification of FosB/ΔFosB-ir area in the cortical ROIs. Runner mice show region-dependent increases in FosB/ΔFosB-ir area (%ROI) in the cortical ROIs. Data are presented as mean ± SEM (n = 5 per group). * *P* < 0.05 vs. Control.

Representative images of FosB/ΔFosB immunostaining in the cortical ROIs are shown in Figure 7. Quantitative analysis revealed region-dependent changes in FosB/ΔFosB immunoreactivity with long-term running (Figure 8). In Runner mice, the FosB/ΔFosB-ir area was significantly higher in the motor cortex (*P* < 0.05) and the somatosensory barrel cortex (*P* < 0.05), but not in the visual cortex (*P* = 0.662) or the olfactory bulb (*P* = 0.523). In the auditory cortex, FosB/ΔFosB-ir area tended toward an increase in Runner mice (*P* = 0.105).

### 5: Neurogenesis

**Figure 9:**
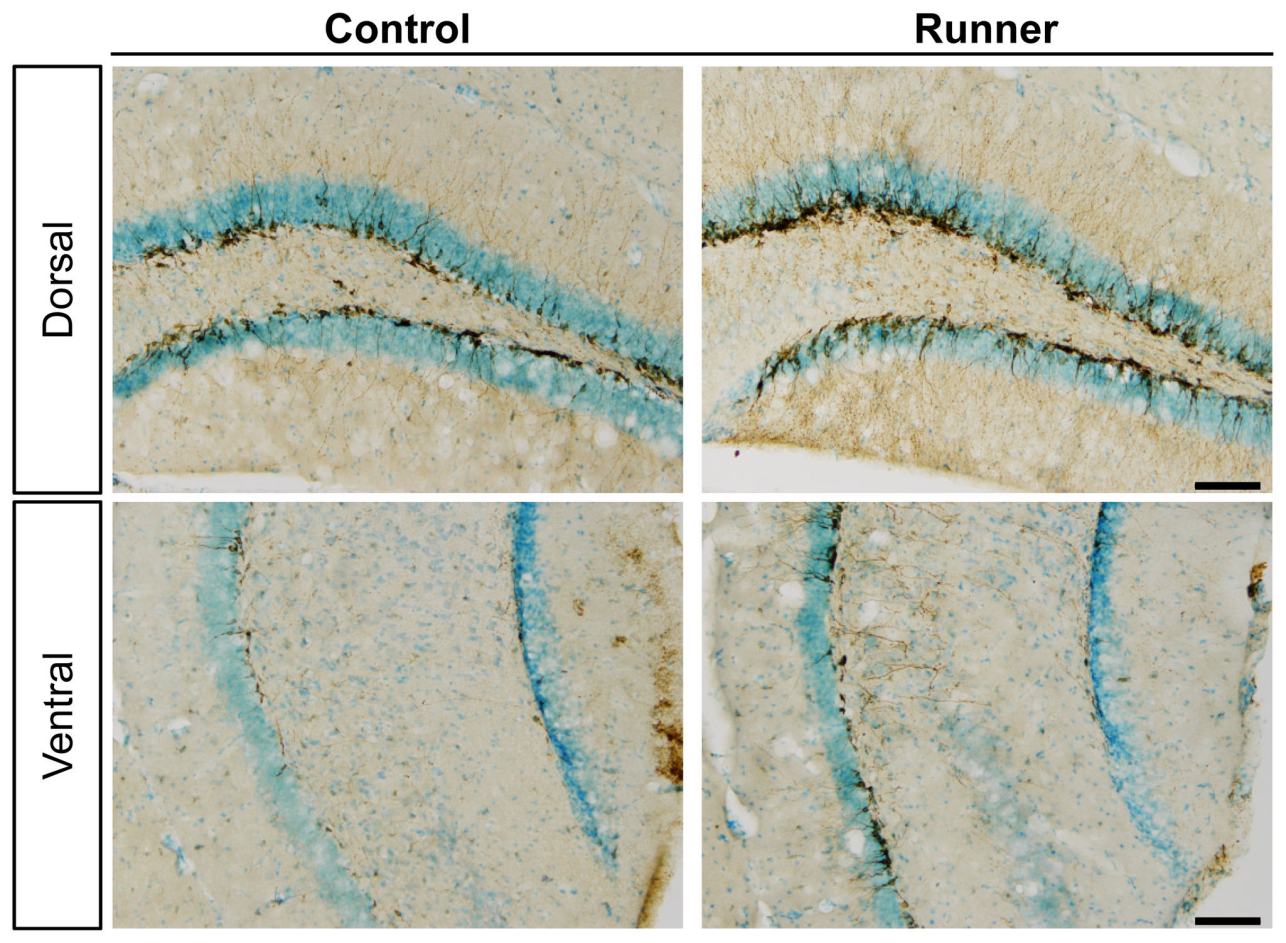
Representative images of DCX-ir immunostaining of the dorsal and ventral DG obtained from the brains of Control and Runner mice, respectively. Scale bar = 100 μm.

**Figure 10:**
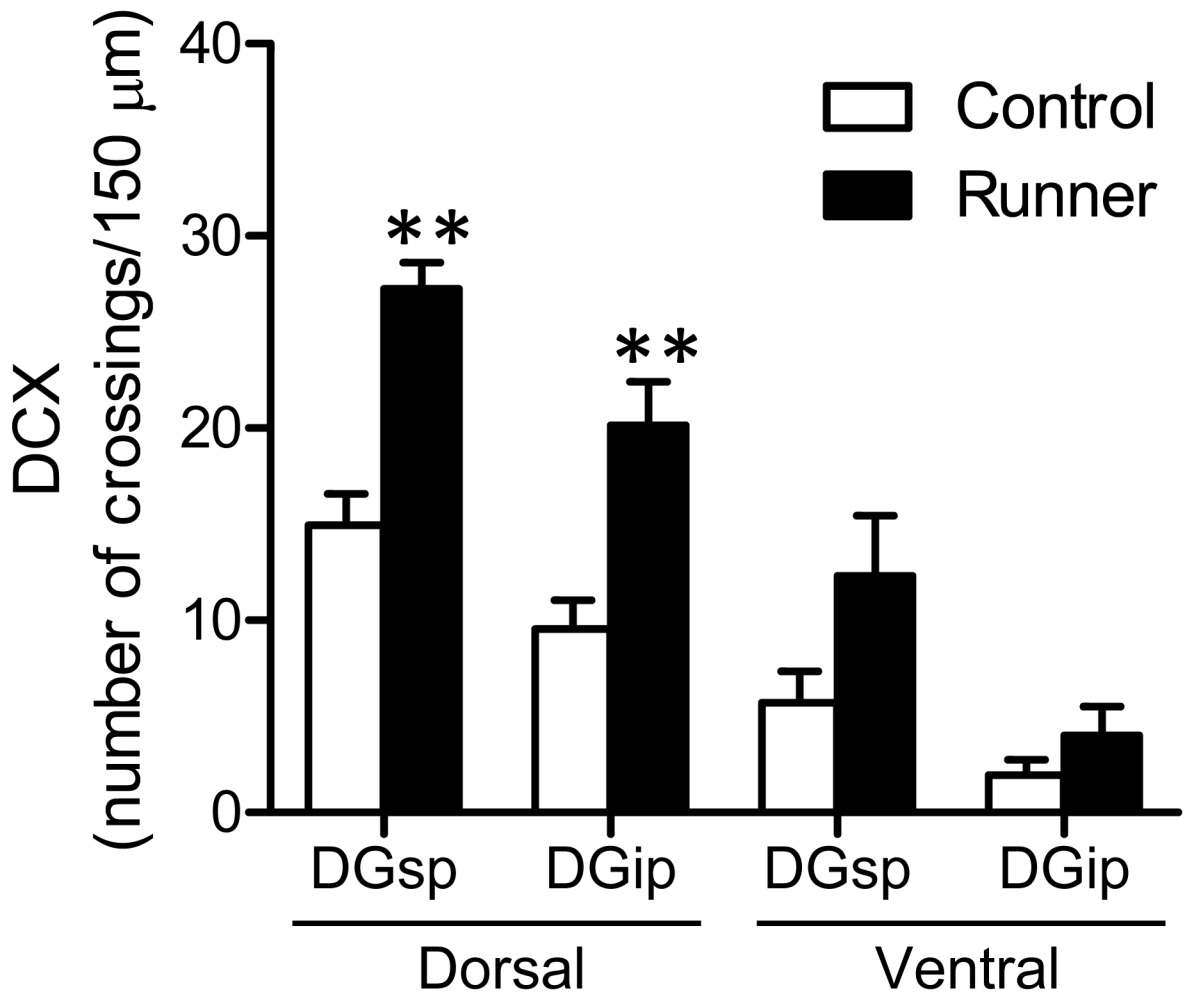
Quantification of DCX-ir immature neurons in the DG. In Runner mice, the number of intersections between DCX-ir dendrites and a line segment was significantly higher in the dorsal DG, but not in the ventral, DG. sp, suprapyramidal blade. ip, infrapyramidal blade. Data are presented as mean ± SEM (n = 5 per group). ** *P* < 0.01 vs. Control.

Representative images of DCX immunostaining are shown in Figure 9. In the dorsal hippocampus, DCX immunoreactivity in Runner mice (Figure 9, right) was qualitatively higher compared to Control mice (Figure 9, left). Compared to the dorsal hippocampus, DCX immunoreactivity in the ventral hippocampus was weaker in both Control and Runner mice. In Runner mice, the number of crossings was significantly higher in the dDGsp (*P* < 0.01) and dDGip (*P* < 0.01; Figure 10). In the ventral hippocampus, the number of crossings in Runner mice was tended to increase, but there were no significant differences between groups (vDGsp, *P* = 0.101; vDGip, *P* = 0.257; Figure 10).

### 6: Correlation between FosB/ΔFosB expression and neurogenesis

**Figure 11:**
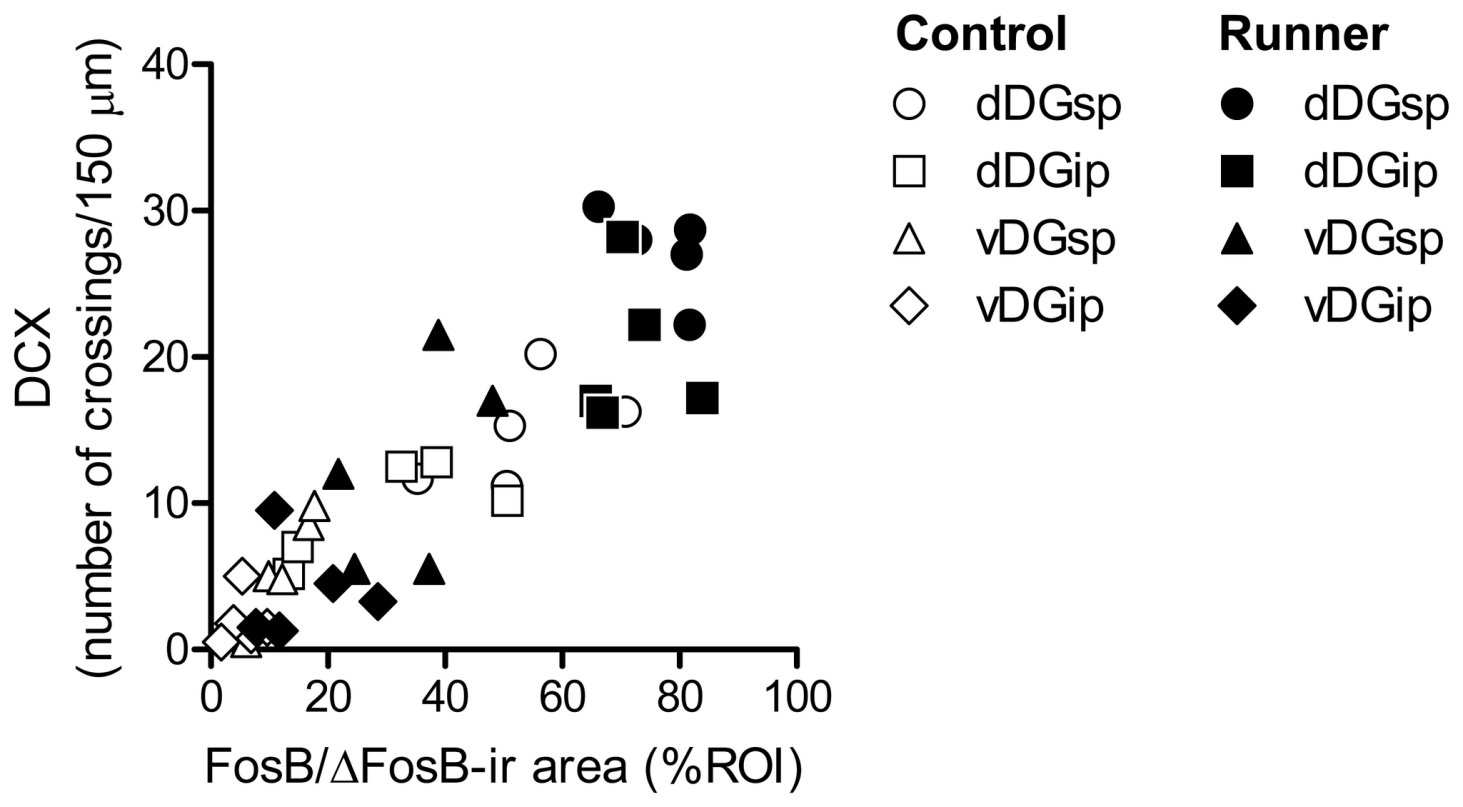
Correlative association between FosB/ΔFosB expression and neurogenesis. A significant correlation was found between FosB/ΔFosB-ir area and the number of DCX crossings in the hippocampus.

A correlation analysis was performed between the FosB/ΔFosB-ir area and the number of DCX crossings (Figure 11). Because each data set (e.g., dorsal DGsp in Control mice) consists of only 5 pairs, the analysis was first performed with all 40 pairs. Intriguingly, there was a significant correlation between the FosB/ΔFosB-ir area and the number of DCX crossings (r = 0.885, *P* < 0.0001). In addition, a significant correlations was also identified when the dorsal DG (r = 0.762, *P* < 0.05) and the ventral DG (r = 0.816, *P* < 0.01) were analyzed separately.

### 7: Identification of the FosB/ΔFosB isoform induced by long-term running

**Figure 12:**
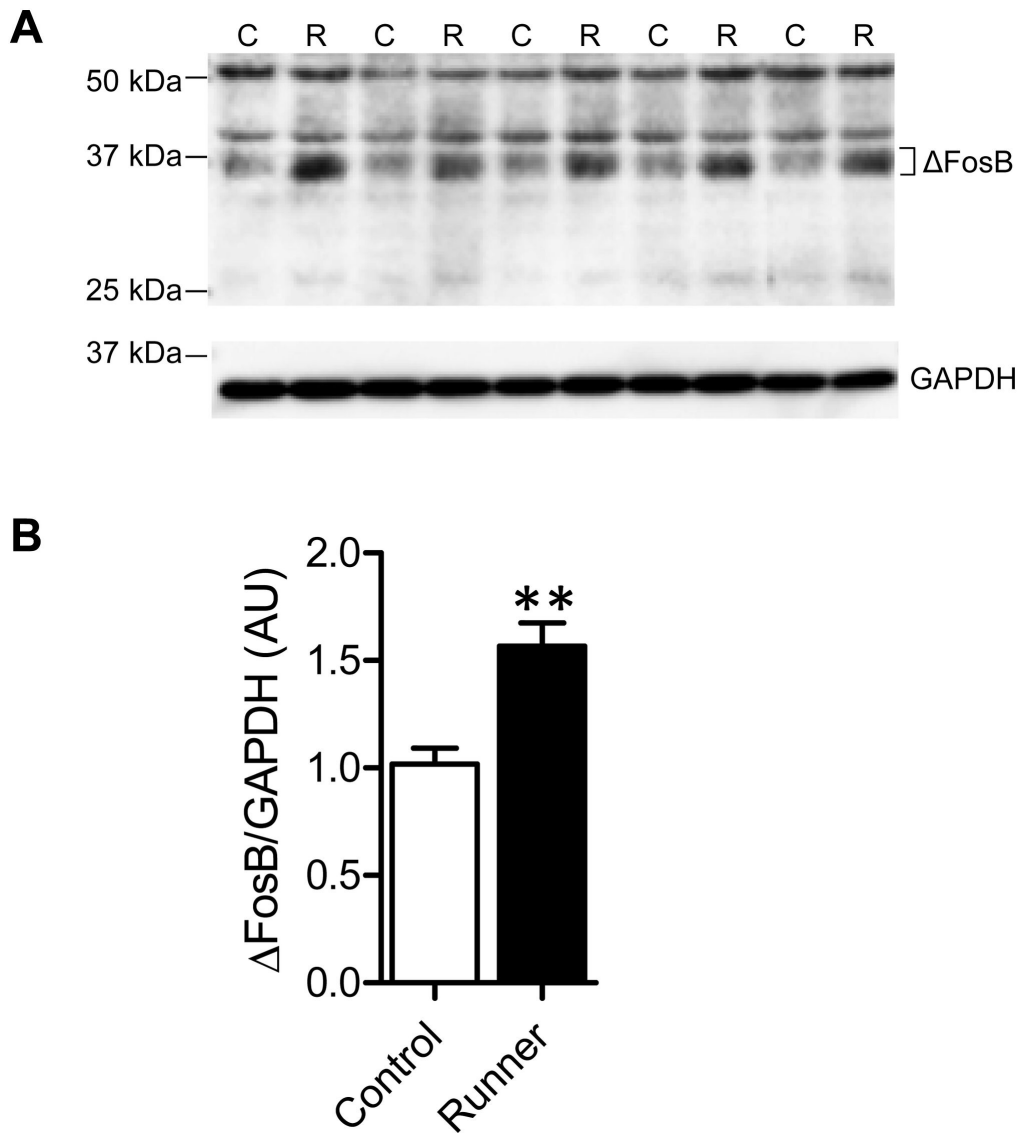
Identification of the isoforms of *the* *fosB* gene product induced by long-term running. (A) A representative western blot using pan-FosB antibody (upper), and anti-GAPDH antibody for a loading control (lower). C, Control mice, R, Runner mice. (B) ΔFosB isoforms (35–37 kDa) were quantified and presented as relative changes from Control mice. Data are mean ± SEM (n = 5 per group). ** *P* < 0.01 vs. Control.

Finally, to identify the isoform of *fosB* gene products induced in the hippocampus in response to long-term running, the hippocampi from an additional cohort of mice were subjected to western blotting with using the same pan-FosB antibody. Multiple bands of 35–37 kDa, representing modified isoforms of ΔFosB [44], were significantly increased in Runner versus Control mice (Figure 12, *P* < 0.01). On the other hand, the 48 kDa FosB isoform was undetectable in either group. Another band faintly visible above 25 kDa likely represents the Δ2ΔFosB isoform (27 kDa). There were two other bands, at above 50 kDa and 37 kDa, which were most likely because of non-specific binding. When quantified, no differences were found in these non-ΔFosB bands between groups (data not shown).

## Discussion

In summary, the present study first performed an immunohistochemical analysis to examining 1) whether long-term voluntary wheel running induces FosB/ΔFosB expression in the hippocampus; and 2) whether a region-specific response exists along its dorso–ventral axis. Four weeks of voluntary wheel running induced a significant increase in FosB/ΔFosB immunoreactivity in all of the hippocampal regions analyzed (i.e., the DG, CA1, and CA3 subfields of both the dorsal and ventral portions of the hippocampus). We confirmed that the 35–37kDa ΔFosB isoform was the major *fosB* gene product accumulating in response to long-term running. These results clearly support the hypothesis that long-term regular exercise is a potent trigger for ΔFosB induction throughout the hippocampus, and that its induction might be a novel molecular mechanism by which exercise affects various types of dorsal and/or ventral hippocampus-dependent functions.

### 1: Validation and limitations of quantifying FosB/ΔFosB immunoreactivity using image thresholding

An image thresholding technique, widely used in immunohistochemical studies for counting the number of target cells and for evaluating cell morphology, was adopted in this study for region-specific quantification of FosB/ΔFosB immunoreactivity [15,45,46]. A significant correlation between the levels of FosB/ΔFosB immunoreactivity quantified by image thresholding and by manual counting was demonstrated (Figure 4). However, because density and overlap prevented counting the number of FosB/ΔFosB-ir nuclei in highly dense areas, the demonstrated correlation only implies accuracy of the image thresholding method when the FosB/ΔFosB-ir areas represents < ~40% of the total ROI area. Therefore, careful interpretation is required for FosB/ΔFosB-ir areas > 40% of the total ROI area.

In particular, in the DG of Runner mice (Figure 4), FosB/ΔFosB expression was greatly induced by wheel running and most of the FosB/ΔFosB-ir nuclei overlapped. In these areas, increased induction of FosB/ΔFosB expression leads to a larger underestimation of the level of expression, regardless of the quantification method used (image thresholding or manual counting). However, despite the risk of underestimation, it is important to note that the present study successfully demonstrated significant increases in FosB/ΔFosB-ir area in the DG of Runner mice. This suggests that the methodological limitations do not compromise our findings. Instead, the potential underestimation increases the reliability of the finding that long-term running increased FosB/ΔFosB immunoreactivity in the hippocampus.

### 2: Uniform Induction of ΔFosB within the hippocampus by long-term running

The hippocampus has anatomical and functional gradients along its longitudinal axis [26], so for the present study FosB/ΔFosB immunoreactivity in the dorsal and ventral portions of the hippocampus was analyzed separately. The data demonstrated that long-term running uniformly increased FosB/ΔFosB expression in all hippocampal ROIs measured. This uniform induction of FosB/ΔFosB immunoreactivity might be non-specifically caused by systemic metabolic changes associated with long-term running. However, it is important to note that there were region-specific increases of FosB/ΔFosB immunoreactivity in the cortex. This result is supported by recent findings showing that an acute bout of treadmill running increased regional cerebral blood flow in the hippocampus, but not in the olfactory bulb [8]. Furthermore, Rhodes et al. (2003) demonstrated that 7 days of voluntary wheel running induced c-Fos expression in the DG and CA2/3 of the hippocampus (CA1 was not measured) and in the sensory cortex, but not in the visual cortex [47]. Taken together, these studies suggest that uniform induction of FosB/ΔFosB expression in the hippocampus is not a non-specific consequence of long-term running. Interestingly, Hawley et al. recently reported that chronic unpredictable stress increased FosB/ΔFosB expression in the dorsal, but not in the ventral, DG of the rat hippocampus [48]. With further investigation, the distinct patterns of FosB/ΔFosB induction such as those elicited by exercise or stress will provide continued insights into stimulus-dependent impacts on the hippocampus.

The primary pan-FosB antibody used in this study is known to recognize all isoforms of FosB proteins. Upon western blotting analysis, we found that the only isoforms that increased in the hippocampus after long-term running were the modified isoforms of ΔFosB (35–37 kDa), the only stable isoforms among Fos family proteins [11]. This finding is in accordance with previous work using pan-Fos antibody to demonstrate that 35–37 kDa ΔFosB is the predominant Fos family protein induced in the frontal cortex by chronic stress [44]. Hence, the increase in hippocampal FosB/ΔFosB immunoreactivity induced here by long-term running most likely reflects the level of ΔFosB.

Less is known about region-specific effects of exercise on molecular and structural aspects of the hippocampus. However, numerous behavioral studies indicate a great potential for exercise-induced improvements in both the dorsal and ventral hippocampal functions. Exercise has been demonstrated to improve spatial learning and memory [34-38] and spatial and contextual processing mainly depends on the dorsal hippocampus [27,28]. In contrast, exercise is also known to exert anxiolytic and antidepressant properties [24,25,38] and these emotional responses are predominantly regulated by the ventral hippocampus [29,30]. The uniform induction of ΔFosB by long-term running seen in this study suggests that some form of neuroplastic changes occurred throughout the entire hippocampus. This would explain why exercise can affect both dorsal and ventral hippocampus-dependent functions.

### 3: Region-specific analysis of exercise-induced neurogenesis

A functional dissociation of neurogenesis between the dorsal and ventral hippocampus has been also receiving increasing attention [49]. In this study, taking advantage of the morphological characteristics of DCX-ir immature neurons [43], we counted the number of intersections between DCX-ir dendrites and a line segment drawn along the middle of the GCL. This measurement did not provide the total number of DCX-ir neurons in the DG, but it enabled region-specific quantification necessary for conducting a correlation analysis with FosB/ΔFosB expression data (see below). Following long-term running, the number of DCX-ir neurons significantly increased in the dorsal, but not the ventral, DG. This suggests that exercise might stimulate neurogenesis more strikingly in the dorsal compared to the ventral portion of the DG. However, previous studies have reported conflicting results in which wheel running increased neurogenesis in both the dorsal and ventral DG [50,51]. In the present study, the number of DCX-ir crossings in the ventral DG tended to increase with running, though the small sample size (5 mice per group) might have limited the ability to detect a statistically significant difference between groups. Therefore, it is likely premature to rule out the possibility that voluntary wheel running can stimulate ventral hippocampal neurogenesis. Further detailed studies are necessary to understand the region-specificity of exercise-induced neurogenesis regarding its multistep process (cell proliferation, differentiation, migration, and survival).

### 4: Functional implications of exercise-induced ΔFosB induction for regulating hippocampal plasticity

Finally, as the first step in recognizing the functional implications of exercise-induced ΔFosB induction in the hippocampus, we examined the relationship of FosB/ΔFosB immunoreactivity to DCX-ir crossings in both the dorsal and ventral DG and found a significant, positive correlation between the two variables. Although the exact mechanisms by which ΔFosB regulates exercise-induced neurogenesis remain uncertain, a recent study demonstrated that *fosB*-null mice, which lack FosB, ΔFosB, and Δ2ΔFosB (all the *fosB* products), exhibited deficits in basal hippocampal neurogenesis, including decreased proliferation of neuronal progenitor cells, increased ectopic migration of newborn neurons, and abnormal DG structures [20]. However, these alterations were not observed in *fosB*(*d/d*) mice, which lack FosB, but not ΔFosB/Δ2ΔFosB. Interestingly, in *fosB*-null mice, expression of some neurogenesis-related genes, including *Vgf* (VGF nerve growth factor inducible) and *Gal* (Galanin prepropeptide) were downregulated [20]. Since VGF and GAL are secretory molecules, one proposal which holds promise considers that neurons expressing ΔFosB may regulate neurogenesis through autocrine/paracrine activity [20].

Additionally, it should be noted that the region where ΔFosB is induced by running spatially overlaps with the region where neurogenic activity is high. This finding suggests that exercise-induced neurogenesis is at the minimum activity-dependent. Neuronal activation is key to maintaining and improving central nervous system function [9], through mechanisms including expression and release of brain-derived neurotrophic factor (BDNF) [52,53], uptake of serum insulin-like growth factor-1 (IGF-1) through the blood-brain barrier [54,55], suppression of apoptosis [56], and regulation of mitochondrial motility [57]. Hence, the present study suggests that long-term exercise triggered repeated neuronal activation, evident in the increased ΔFosB expression, which contributes to enhancing hippocampal plasticity, potentially through these multiple mechanisms described above.

The present study only assessed exercise-induced neurogenesis and its association with FosB/ΔFosB expression in the DG. However, FosB/ΔFosB immunoreactivity was also induced in the CA1 and CA3 subfields. While further studies are required to gain more understanding of the functional roles of exercise-induced ΔFosB expression within these subfields, previous literature offers a promising possibility. Guan et al. (2011) demonstrated that specific ablation of the cyclin-dependent kinase 5 (Cdk5) in the CA1 or CA3 pyramidal neurons impaired memory consolidation or retrieval, respectively [58]. Interestingly, the Cdk5 is the downstream target of ΔFosB [59] and is involved in regulating synaptic plasticity [60]. Therefore, exercise-induced ΔFosB expression could be involved in regulating synaptic plasticity through Cdk5 activation in the CA1 and CA3 subfields.

## Conclusion

While acute bouts of exercise were known to induce the expression of immediate early gene proteins in the hippocampus, the present study provides the first evidence that long-term regular exercise significantly induces ΔFosB expression in the entire hippocampus. This uniform induction of ΔFosB supports the current understanding that exercise is an effective non-pharmacological intervention able to improve multiple hippocampal functions. Together with the significant correlation between FosB/ΔFosB expression and neurogenesis, these data are provocative and indicate a need for further studies delineating the role of ΔFosB in mediating the effects of exercise on hippocampal function, including neurogenesis.
